# Reorganization of the Tennessee State Dental Association

**Published:** 1911-10-15

**Authors:** Arthur D. Black

**Affiliations:** Chicago, Ill.


### REORGANIZATION OF THE TENNESSEE STATE DENTAL ASSOCIATION.

BY ARTHUR D. BLACK, D.D.S., CHICAGO, ILL.

Mr. President and members of the Tennessee Society: I am not really sufficiently familiar with the exact conditions in this state to discuss in a positive way plans of reorganization which might be best for you to adopt. I think I might, however, in a very few minutes, tell you rather a little something of what has been accomplished in Illinois as a result of our re-organization, and possibly a word or two by way of suggestion as to what might be the best plan for you to follow here.

First a word regarding membership. In Illinois in 1904 we had in our society about 400 members; of those in that year 274 actually paid dues, our dues then being $5.00 We re-organized during that year, and in 1905 we had a paid membership of 1,260 as compared with 274 the year previous. In 1906 we had a little over 1,300; in 1907 we had a little over 1,500, in 1908 we had a little over 1,600, and in 1909 a little over 1,700. This year our membership will probably show very close to 1,800 members, if not a little above that, so that from a standpoint of membership alone there can be no possible doubt of the success of this plan with us, and the success of it in other states that have adopted it has been similar to ours. Naturally in a city like Chicago it is difficult to get a percentage that will compare with other communities. There was a good deal of question in the minds of a good many, especially some of our older members, whether or not the Society could afford from a financial standpoint to take this step, when the dues were dropped from five dollars to two dollars, and it was a question whether with the additional expenses we would have to carry on the organization as a result of this re-organization within the first year; but we found that we had not only enough money to carry out the Association work and pay all the expenses of organization, but still have more money in the treasury than we ever had before. At our meeting this year our treasurer's report showed a surplus of nearly two thousand dollars in the treasury. However, the matter of membership alone is not the most important item in this matter. What you wish more than anything else is to forward dentistry in your state, and I think you will have to agree that a comparatively few men, say if you have thirty per cent. of the dentists in your state, you are hardly entitled to say that you represent dentistry in your state with so few members; you ought to have at least half of the dentists in this to make a truly representative organization, and the state society ought to be able to direct the thought and action of the dentists who are practicing in your state

The strongest feature of this plan of re-organization is the local or district society, the organization which will bring the men of a community who are fighting the same battles, who have the same things to contend with,—which will bring these men together and get them well acquainted.

First, I will mention the fact that the fees that are paid for dentistry in the state of Illinois add up to-day somewhere between four hundred and five hundred thousand dollars a year more than they did previous to our re-organization. That is purely an incidental. But it is not the dental profession alone that has profited by that increase in fees, but the people have profited more than the dentists, because of the much better dental service that has been given for that increased fee. I will state to you that in any number of communities in the state of Illinois where the price paid for a Richmond crown, the average price, was five dollars or a little less before we organized; to-day the ordinary price paid for a Richmond crown in that same section is twelve dollars, and the people are getting more nearly twelve dollars worth to-day than they were five dollars before, because they are getting better dentistry, and both of these things have been the result of our organization. The dentists understand each other better and become better acquainted. Now the increase in fees has been brought about largely without any paper having been read in a local society, but as one of the results of the men being better acquianted so that each man really knew something about what the other man was charging.

We have twenty-eight local or district societies in the state of Illinois.

By looking over the map of the state of Tennessee you ought to re-organize your state on a plan a little different from ours. It would be better for you to follow more nearly the plan of the state of Nebraska. There are sections in Nebraska that are very sparsely settled where the dentists are scattered over a pretty wide territory and it will take the dentists in probably eight or ten towns to make up a local society. In Nebraska thay have divided their state up into six districts, and they have one local society in each district. I am inclined to think that is the better plan for Tennessee, because some sections of this state are not thickly settled. You ought to have the districts to cover a large territory so that you will have some fifty or seventy-five dentists that live in that territory, rather than try to make too many small societies. However, you can adopt part of each plan. In one section of the state where there are enough dentists to make a society that would have jurisdiction over a small territory, that can be done; and in other sections they might be scattered over a very much larger territory.

There is one phase of this re-organization scheme which came up a great many times with us, and that has been the proposition, as some men have put it, although wrongly—of requiring a man to join the state society because he joins his local association. Now the man that takes that view does not comprehend this re-organization proposition; it should be understood that the local society in any section, or the district society, is the state society in that section. You do not join the local society at all, you join the state society, and that local society is simply that part of the state society which happens to be in that part of the state. It places the matter of electing men to the state society in the hands of men who know the men who want to get in, and this prevents the election of those who are undesirable, but in the organization and conduct of our twenty-eight local societies in our state, we have not had any trouble of that kind, and I do not think you would have in Tennessee. I think most all of you appreciate the fact that we want to get all the good men in these societies that we can, and we have not had any trouble. The state dental society organized on this plan gives you a weapon for good in any cause that you cannot appreciate until you get into it. I might cite the passage of our last dental law in Illinois, and I might say primarily that we passed it. The first dental law was passed in 1880 and we have been almost constantly before the legislature after that and there was no dental law passed in the state of Illinois from 1880 until 1905. We passed a new law in 1905, another one in 1907, and another one in 1909, we passed a new law at all three sessions of the legislature and we carried them through almost without a dissenting vote in either house. Just to cite one example of the way in which the association works; when one of these laws was passed our governor said he thought he would veto it. When asked why he showed a stack of letters from dentists over the state asking that it should not be passed, because we had some restrictions in the bill on ethical practitioners and the outsiders did not want it passed. We said just to give us forty-eight hours and we would show that there were more dentists in favor of it than there were against it. We had the officers of our local societies primed up for it and we landed over three thousand letters and telegrams in the governor's office inside of forty-eight hours, all of which were asking that the bill be passed. So when you can get results like that you are accomplishing something. And we have been just as successful in matters of oral hygiene in all public matters in getting something adopted in the text books of the public schools for the good of the people of that state. If your committee will study this proposition carefully and decide upon a good plan for you to adopt in this state, I am sure you will never regret having done so. I think you ought to consider this proposition as an entirely separate one from the present organization throughout the state, because I think you might accomplish something in that way, you ought not to have to wait on the National because you might all die before they get to you.

DISCUSSION.

DR. LEONARD: As has been suggested by Dr. Black, the primary work of an organization of this kind is to get a more representative membership from the members of the profession and raise the status of dentistry throughout the state. Besides increasing the membership of our state society it will enable us to do better work through the other organizations of which we would be and become a part. This is the plan of organization that has been adopted by the American Medical Association and which has been eminently successful in every way. Now I think that it is a plan that would certainly result in a magnificent work for our state and for every member of the profession in the state. I will suggest and make that as a motion, that a committee of three be appointed at this meeting, to report to-morrow on some definite plan of action—some plan that will look to this work being done during the next twelve months, before another meeting comes about; this committee to make some estimate as to the probable number of district societies that would be feasible, and an estimate probably as to the appropriation that would be necessary. I would favor certainly an appropriation from this society at this present meeting to meet such expenses as would be absolutely necessary to perfect this organization, or at least to begin it and do such work as we could do through the next twelve months. This committee might go over the map of the state and arrange, without any very definite form, just a sort of tentative plan to be presented to the society, and that would contemplate the expenditure of a certain amount of money, and then a permanent committee be appointed to take this matter up and work in conjunction with permanent members of the state association throughout the state and with the men who live in these districts, in an effort to perfect an organization if possible before the next year. We can organize these district societies and have them ready by the time of the next meeting when we can perfect the whole organization. Motion seconded.

Dr. Henry W. Morgan: Mr. President, I would like to inquire if that was not acted on a while ago by the committee on President's address?

Dr. Leonard: That was a recommendation from the committee for a committee to be appointed, but this committee was to make plans to begin the work perhaps next year. This motion of mine contemplates the beginning of the work immediately. Motion put and carried unanimously.

